# Balanced diet and daily calorie consumption: Consumer attitude during the COVID-19 pandemic from an emerging economy

**DOI:** 10.1371/journal.pone.0270843

**Published:** 2022-08-04

**Authors:** Ashutosh Kolte, Yogesh Mahajan, László Vasa

**Affiliations:** 1 Department of Management Sciences, Savitribai Phule Pune University, Pune, India; 2 Symbiosis Centre for Management and Human Resource Development, Symbiosis International University, Pune, India; 3 Széchenyi István University, Győr, Hungary; University of Jyvaskyla, FINLAND

## Abstract

This article tries to explore consumer attitudes regarding a balanced diet and daily calorie intake monitoring during the COVID-19 pandemic in India. It has become vital to boost people’s immunity because of reoccurring diseases such as COVID-19, Ebola, and other chronic diseases such as diabetes, thyroid disease, etc. Healthy diets are important for supporting immune systems and keeping track of daily calorie consumption is an accompaniment to this. The research on attitudes toward a balanced diet is reviewed in this empirical study. Researchers employed a tri-component attitude model to assess consumer attitudes about a balanced diet and to track daily calorie consumption. A sample of 400 respondents was surveyed and data were collected with a structured questionnaire. The data were analysed using the structural equation modelling technique. The majority of respondents were found to lack declarative knowledge of both a balanced diet and daily calorie consumption. The effects of the COVID-19 pandemic on consumer attitudes about a healthy diet and daily calorie intake were effectively evaluated using beliefs, affection, and intentions. The repercussions for the government and business community were discussed. This study also evaluates the usefulness of the tri-component attitude model in the Indian context.

## 1. Introduction

Consuming a balanced diet is an ardent task for most people in the world. Due to recurring diseases like COVID-19, Ebola, Swine flu, and chronic diseases like diabetes, hypertension, thyroid, etc., it has been increasingly necessary to improve people’s immunity. Immunity helps to fight these diseases and other ailments such as blood pressure, diabetes, thyroid, etc. Immunity can be increased by consuming a balanced diet and monitoring daily calorie consumption [1].

The impact of COVID-19 has shaken the world to its roots—globally, more than 165 million people have been infected, and there have been more than 3 million deaths as of this time [2]. More than 43 million people have been infected in India with over 521,000 deaths reported [2]. The everyday lives of people are disturbed through, for example, closure of public places like malls, gardens, cinema theatres, etc., in order to curb the spread of COVID-19. In this challenging situation, it is necessary to maintain health and improve immunity. In order to boost immunity in quarantined patients in India, properly balanced diets are provided to aid in fighting COVID-19 in addition to treatments and vaccines. Such is the effect of this pandemic that the economic growth rate of many countries has declined, and many people, globally, have lost their jobs and enterprises have ceased trading.

58 percent of the 57 million annual deaths in the world are estimated to be due to Non-Communicable Diseases (NCD); mostly related to the heart, chronic pulmonary disease, cancer, and diabetes [3]. A poor intake of fruit and vegetables, high blood pressure, high blood cholesterol levels, overweight or obesity, physical inactivity, and nicotine use are the most significant risk factors for NCD. In the next few decades, population growth and aging are expected to significantly increase in conjunction with economic transformation and resulting shifts in jobs, and environmental risk factors [4]. People suffering from the aforementioned diseases and older people are among the most vulnerable to recurring diseases like COVID-19, Ebola, etc. COVID-19 was declared a global pandemic by the World Health Organization (WHO) in 2020.

**Fig 1:**
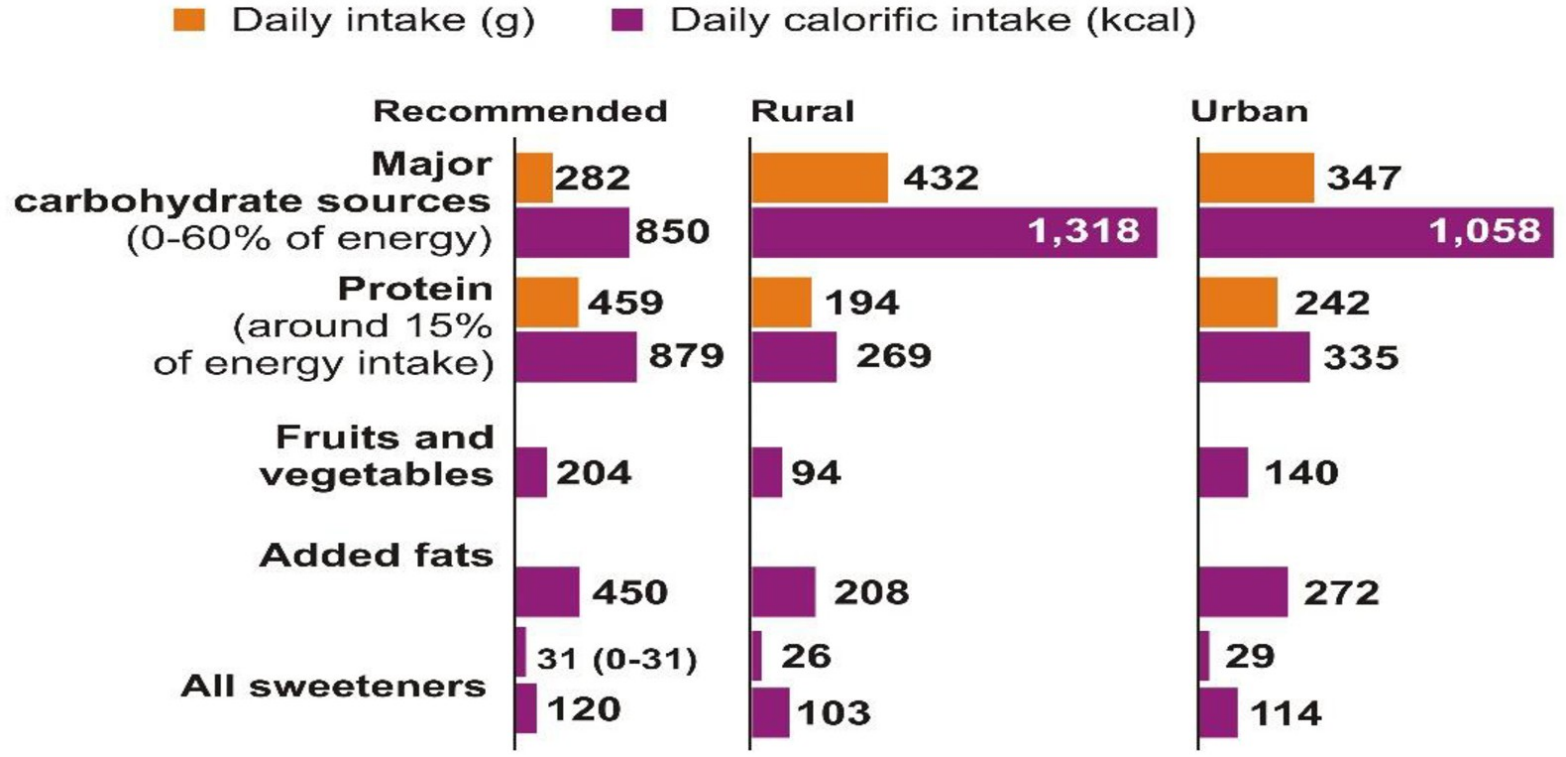
Daily calorie intake. Source: [7]. *Quantities not mentioned for some categories as data for some individual components was in terms of number of packets/cups etc.

The likelihood of death due to COVID-19 is high if people already have non-communicable diseases [5]. In a developing country such as India, the risk of COVID-19 is very high. However proper consumption of a balanced diet and monitoring daily calorie intake [6] are a necessity in times of a pandemic. According to Lancet, the average Indian (urban or rural) consumes a higher amount of carbohydrates in the form of cereals (Fig 1) than The Lancet commission [7] recommends. Whereas the protein consumed is significantly less than recommended by the average Indian, the daily calorie intake in both rural (2214 kcal) and urban (2169 kcal) India is less than the reference diet (2,503 kcal/capita/day), except for the wealthiest 5 percent of the population [8].

**Fig 2:**
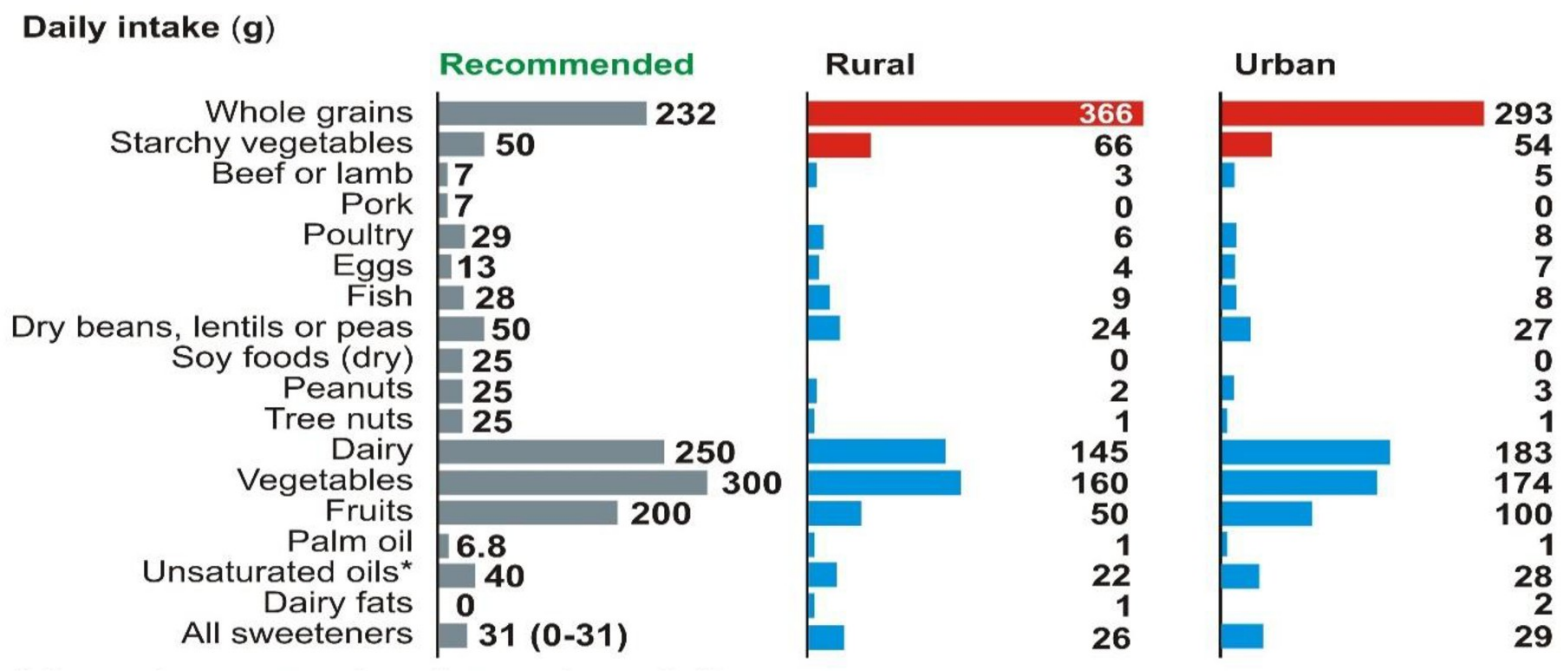
Daily food intake. (Source: EAT-Lancet commission) (*olive, soybean, rapeseed, sunflower, and peanut oil) Note: Whole grains refer to rice, wheat and wheat products, millet and their products, cereal products refer to maida and other refined products from cereals, palm oil refers to vanaspati, unsaturated oils refer to sunflower, groundnut and refined vegetable oils, dairy fats refer to ghee and butter.

In addition, there is less fruit and vegetable intake by Indians and too little unsaturated oil consumption (Fig 2). With an emphasis on natural products over packaged food, a diverse diet was recommended by the EAT-Lancet committee [7].

A population must have sound knowledge and a positive attitude towards a balanced diet and daily calorie consumption [9]. This study investigated the declarative knowledge and consumer attitude towards a balanced diet and monitoring daily calorie intake among the working population in India by using a tri-component attitude model during the COVID-19 pandemic.

## 2. Review of literature

Nutritional guidelines were announced during the COVID-19 epidemic emphasizing the importance of a balanced diet to sustain a well-functioning immune system and prevent or reduce chronic diseases and infections. Dietary guidelines from WHO during the COVID-19 pandemic are comparatively higher than the standard dietary recommendations by the WHO [10]. Literature shows clear evidence of an inadequate diet and the risk to health thereof [11]. Research from Boylan that linked a poor diet and a lack of physical activities together with the risk of obesity has helped create and promote recommendations to increase public health [11]. Over-consumption, lifestyle, accessibility to food and strong pressure exerted by the foodstuff industry have disrupted all elements of food consumers’ behaviour which have caused them to lose out on nutritious milestones that have been achieved by family education [12, 13]. Consumers are not initially looking for seasonal foods, but for what seems to be the most favourable value for money. In this sense, more than ever, the need for knowledge to connect nutrition and wellbeing, to avoid nutritional and metabolic disorders, and the need for the fundamental values of a healthy diet to mainly be adopted by a new generation of customers. They have positive expectations towards quality of life and a special perception of well-being factors. Limitations brought about by pandemic, particularly, voluntary and involuntary online studying, have an essential impact on the lifestyle and health condition and became an important feature of well-being. Health is understood to depend on the diet to a large extent [14]. Many authors have stressed the importance of a balanced diet and daily calorie consumption in daily life [15]. Also, experiments have shown that a balanced diet helps to reduce weight among obese people [16]. Nutritional education among consumers helps increase awareness about a balanced diet and daily calorie intake [17].

Knowledge does not consist of a single structure but different components. JR Anderson first identified two elements of knowledge. One representing ‘to know’ and the other representing ‘to know how’. These principles are applied to the concepts of procedural and declarative knowledge in cognitive psychology. Knowledge of facts and objects is called declarative knowledge, while knowledge of performing actions is procedural knowledge [18]. Procedural knowledge is also closer to behaviour. These differences in declarative and procedural knowledge are extended in the nutrition domain [19]. Few studies have evaluated declarative knowledge [20, 21]. In contrast, some have included concerns about procedural knowledge, typically in the form of food options or by posing a query [22–24]. Declarative knowledge among the population can be ascertained through the study of consumer attitudes towards a balanced diet and daily calorie consumption. Consumer attitudes and perception play an important part in the dietary behavior of an individual. Attitude plays a key role in forecasting behavior, and persuasive communications are undertaken to help people partake in healthy behaviour. Most diet awareness initiatives for subpopulations such as cardiac patients [22], adolescents [23], or young men [25] are planned and applied.

### 2.1 Tri-Component attitude model

**Fig 3:**
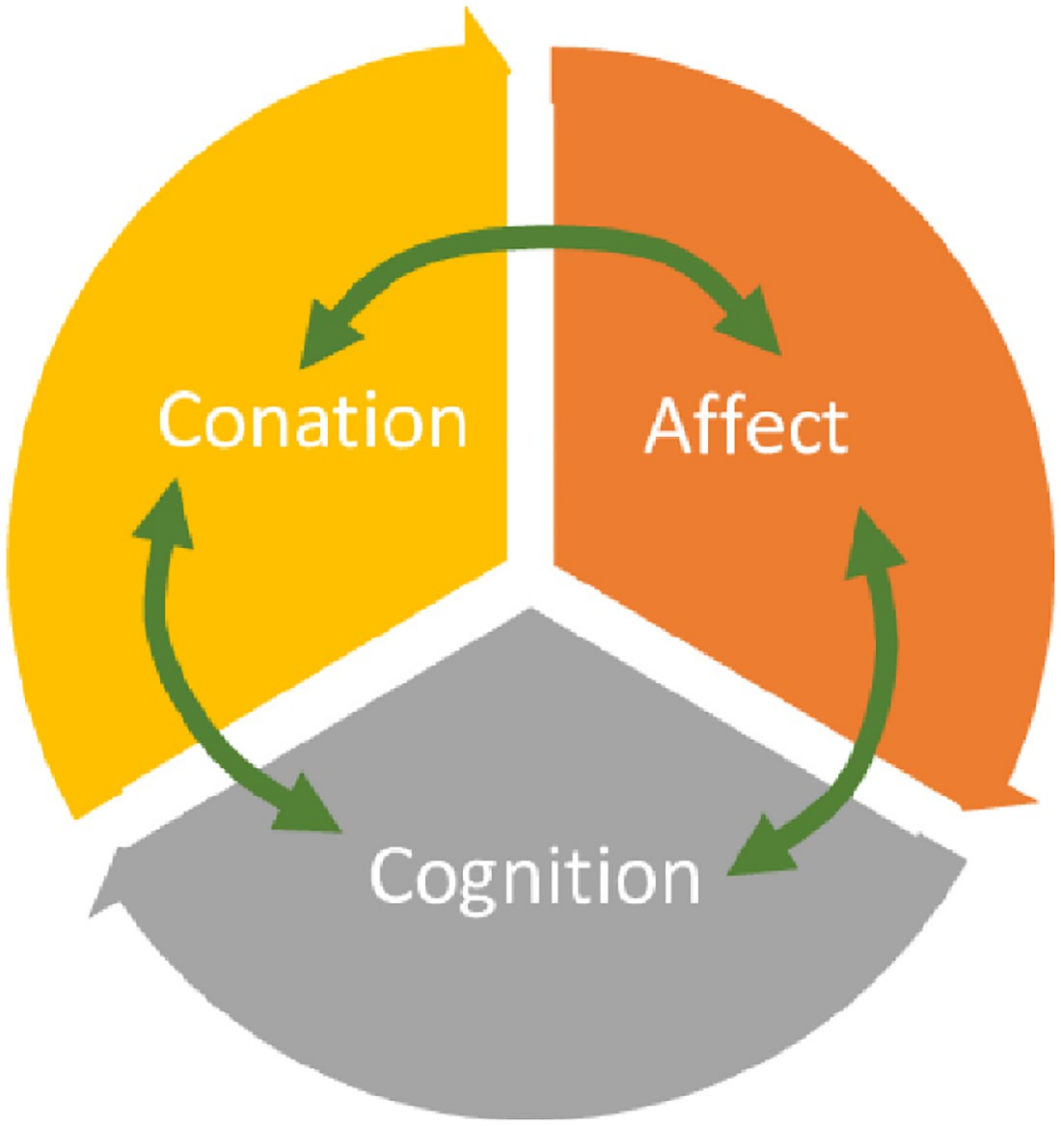
Tri-component attitude model. (Source: [28]).

Psychologists have come up with models and theories that help understand consumer attitudes; these assist in understanding consumer buying behavior [26, 27]. Important models or theories of consumer attitudes discussed in the literature include the theory of planned behavior, tri-component attitude model, the hierarchy of effects model, theory of reasoned action, and attitude toward behaviour model. This model, regarding components of attitude and their relationships, provides varying perspectives. The tri-component attitude model is the fundamental model to study the attitude of consumers developed by [27]. The tri-component attitude model (Fig 3) has three constructs: viz, cognition, affect, and conation [28] as shown in Fig 1. Arnould [29] proposed that this model is labeled as the ABC model, with affection, beliefs, and conation as rearranged components. Cognition (understanding) of a person is the consumer’s perceptions and knowledge through understanding the related item and facts from several sources [28, 30].

The conative component, the way the attitude we have influences how we act or behave, describes the intention to buy or follow, which is also known as behavioural intention. Some explanations consider conation as the behaviour itself [28]. However, Assael [30] conflicts with this view. He asserts that the beliefs and assessments of a specific item sometimes need not change the intention of consumers to purchase or follow, especially if economic incentives are sufficient; such as a sharp price decrease. In developing a marketing strategy [31], it is essential to measure behavioral intention. Studies have been carried out regarding dietary behavior during normal times but such studies during the COVID-19 pandemic have not been carried out. For instance, Popescu and co-authors undertook exploratory research based on the methodology of the food diary by evaluating the features of Romanian food consumption for the next decade. Investigators analyzed the respondent’s diet’s medium energy use and macronutrients to identify possible dietary imbalances [13]. Consumers’ healthy eating choices are strongly linked to epistemic and emotional values [32].

In Mexico, Carrete researched the perception of drivers and inhibitors of sound dietary behaviors, with attention paid to serious problems with obesity [33]. The theoretical basis for this research was the theory of planned behavior (TPB) and protection motivation theory (PMT). Results show that poor self-efficacy and high costs inhibit behavioral change [33]. Sanusi studied the dietary behavior of university students and noticed that attitudes toward behavior was found to be the more influential predictor of intention to use dietary guidelines in Malaysia [34]. Worsley concluded that nutritional knowledge is a necessary factor in improving customer behavior, but it is not adequate. For nutritional promoters, the interplay of motivational and information processing factors and the distinction between declaratory and procedural knowledge is essential [19, 35]. Additionally, research on monitoring daily calorie intake has shown positive results. Alamer found that customers who checked the daily calorie intake were more likely to change their attitude towards eating behavior [36]. Researchers have used cognitive, affective, and conative components of consumer attitudes to study dietary behavior in the literature in normal times. For example, Sijtsema [37] examined the nature, structure, and use of projective approaches in cognitive and affective terms (expressive and associative) to discover consumers’ health perceptions. They observed that participants associated healthy living with feeling comfortable and satisfied and found an equilibrium between being active and passive [37]. Dietary education is related to maximum scores for food knowledge in developed countries [38].

The consumer’s attitude was not assessed coherently despite its significance in marketing. For evaluating the dimensionality, reliability, and validity of customer attitudes to imported poultry products, Makanyeza measured the assessment of consumer attitudes based on a tri-component attitude model [39]. The study showed that the customer approach to poultry products has 3 constructs: viz., cognition, emotions, and purchase intentions [39]. In consumer health habits, Makarem studied emotion and cognition. He found that wellness programs aimed at improving treatment compliance should consider alternatives to messages demanding control over patients’ health [40]. In national surveys of a member state of the European Union, Authors studied the attitudes of adult individuals to nutrition, food, and health. Most EU customers do not think they need to change their diets because they believe they are safe enough (Erdeiné Késmárki-Gally and Fenyvesi, 2012). For certain classes of the population, prices may play a large part in choosing food in Europe as in other developed and developing regions. Therefore, the issue with nutritious food proponents in the EU may not be a lack of awareness, but rather of the nature of diet recommendations [41].

On the other hand, in Asia, dietary behavior is influenced by Western culture. Authors observed that Koreans preferred Westernized and unbalanced foods, providing fewer nutrients [42]. It was underlined that the growing demand for Western-type foodstuffs can be the implication of the general popularity of the American lifestyle in South Korea. Moreover, the increasing income of family households—especially in the younger generation—generated a demand for food products that used to be almost unknown in the traditional Korean diet. The Korean Medical Association issued 3 top methods for a safe and healthy diet in 2017 to reverse this unfavorable cycle. These recommendations were intended to enable the population to consume a healthy diet that matches the prescribed intake of calories and reduces the threat of obesity as well as diseases associated with an unhealthy diet. Avoiding over-eating and being physically active at a young age can help reduce these conditions [42]. Whilst a balanced diet should be followed by consuming a range of foods, the ideal is different from what people consume. Food rich in nutrients, including food supplements and fortified foods, may be used as a powerful means to guarantee micronutrient satisfaction in persons at all phases of life and with shifting lifestyles, where food selection can incorporate an optimal diet [43].

Thomas examined the connection between food preferences and the understanding of a balanced diet of primary school students. There was so significant connection between the pupils’ diet selection and their knowledge of a balanced diet. The findings revealed that children make ’good decisions’ but need the awareness behind these healthy choices to change their approach to a balanced diet [44]. Casini and co-authors analyzed the development of dietary habits in Generation X. The findings indicate that dietary lifestyles with high animal protein content are strengthened, especially for consumers with poor educational levels. Convenience foods become essential, particularly for couples and for families with children. The significance of consumption at home, mainly among individual men, is increasing [45]. The food industry should ensure that the messages it has created are inspiring and meaningful for consumers’ lives by ongoing dialog with its consumers and studies on consumer knowledge and dietary attitudes [46].

For urban American youth, Fila studied their healthy eating behaviour. He found no association between healthy eating and intentions. A balanced food supply and taste were the most predictive obstacles. The behaviour of boys was predetermined by subjective norms, while the behavior of girls by obstacles is more expected. Results show that healthier eating activities should concentrate on working with families to make healthy food attractive for young people [47]. Song classified products that consumers think are safe and harmful and examined how these products affect consumers’ attitudes [48].

Research findings demonstrate there is a difference between product appeal dependent on outstanding sensory properties shown by young customers and the actual dietary content of products, transforming into unbalanced nutrient profiles in packed food. Research results show that young customers need to be reassured that delicious food is not always nutritious and to have proper nutrition and a healthy way of life is of value. Young people should be better trained in dietary methods, weight control and fitness [49]. It is found that consumers perceive functional foods help them follow a balanced diet but are anxious about labels communicating the health benefits [50].

## 3. Research methodology

Various methodologies such as projective techniques, questionnaires, focus groups, and images of food are used by researchers to study the attitude and perception of respondents for a healthy diet and daily calorie intake in the literature [44]. Focus groups and questionnaires are also used for pilot studies in the literature. This study is empirical. As this study was conducted during the COVID-19 pandemic, physical data collection was not possible due to lockdown. However, using an online questionnaire prepared for the sole purpose of measuring consumer attitudes towards a balanced diet and monitoring daily calorie intake, we were able to collect data in India between March, 2020 and May, 2021. The pilot study involved sending the questionnaire to 20 people to refine the constructs for the study. A structured questionnaire [25] was then prepared and used to collect data from respondents. Researchers explained through a write-up to each respondent that the purpose of the survey was to find out how they feel about a balanced diet and monitor daily calorie intake. Consent was obtained from each respondent before asking him or her to fill out the questionnaire. The convenience sampling method was used to collect the data from respondents.

The questions were structured on the tri-component attitude model to take into account attitudes such as cognition, affection, and conation [30]. The scales used by various authors like [28, 51, 52] were considered to develop a holistic scale to understand the attitude towards a balanced diet and daily calorie intake. Measurement of 3 components (constructs) was carried out by 12 items (see Table 1). The 5-point ordinal Likert scale was used to measure the 3 components with: Strongly agree (1) to Strongly disagree (5). The items F1-F4 were used to measure cognition (beliefs), items F5-F8 were used to measure affection (feelings), and items F9-F12 were used to measure conation (intentions) of the consumer.

**Table 1:** Items of attitude.

Items	Name
Consumption of a balanced diet is beneficial for good health	F1
Monitoring daily calorie consumption is beneficial for good health	F2
Consuming a balanced diet will reduce the threat of infection of COVID-19	F3
Monitoring daily calorie consumption will reduce the threat of infection of COVID-19	F4
Eating a balanced diet will make me feel happy	F5
Monitoring daily calorie consumption will make me feel happy	F6
I will become stronger by consuming a balanced diet	F7
I will become stronger by monitoring my daily calorie intake	F8
I will consume a balanced diet on daily basis due to the threat of the COVID-19 pandemic	F9
I will monitor my daily calorie consumption due to the threat of the COVID-19 pandemic	F10
I will recommend others to consume a balanced diet because of the COVID-19 pandemic	F11
I will recommend others to monitor daily calorie intake because of the COVID-19 pandemic	F12

(Source: own development).

The terminology adopted is based on work done to quantify customer beliefs in a balanced diet and daily calorie consumption [52]. Consumer emotion measurement is based on the work of Bruner and his co-authors [51]. To measure consumer intentions, the recommendations of [52] were followed. An online questionnaire using Google Forms was sent to more than 2,000 respondents via E-Mail and WhatsApp, out of which 412 responded to the questionnaire. 12 responses were not considered due to inadequate data from the respondents. A sample of 400 was considered for this study as per the statistical formula for calculating sample sizes for online surveys. Only respondents above the age of 25 years were selected for this survey. The purpose of this selection was to investigate the attitude of the working population towards a balanced diet and daily calorie intake. The study uses the tri-component attitude model to study the attitude and perception of respondents towards a balanced diet and monitoring daily calorie intake.

Based on the above comprehensive literature review, the following hypothesis was formulated. The hypothesis was developed for both a balanced diet and daily calorie consumption. The hypothesis in the case of balanced diet and monitoring daily calorie intake is:

H1: Consuming a balanced diet is positively associated with the benefits of good health
H2: Monitoring daily calorie intake is positively associated with the benefits of good health
H3: Monitoring daily calorie intake is positively associated with reducing the threat of infection of COVID-19
H4: Consumption of a balanced diet is positively associated with happiness
H5: Monitoring daily calorie intake is positively associated with happiness
H6: Consuming a balanced diet is positively associated with a stronger self in the COVID-19 pandemic
H7: Monitoring daily calorie intake is positively associated with stronger self in the COVID-19 pandemic
H8: Consuming a balanced diet is positively associated with intentions due to the threat of COVID-19 has become a necessity
H9: Recommendation to others to consume a balanced diet is positively associated with intentions due to the threat of COVID-19 pandemic
H10: Recommendation to others to monitor daily calorie intake is positively associated with intentions due to the threat of COVID-19 pandemic

## 4. Data analysis and results

Data cleaning was executed and then coded in JASP software [53] and entered. Exploratory factor analyses were conducted in JASP software to confirm the elements underlying the customer attitude construct. The reliability of the three components’ measurement scales was checked using Cronbach’s alpha. Finally, the JASP program conducted a confirmatory factor analysis and structural equation modelling.

### 4.1 Sample profile

Collected data illustrates consumer attitudes towards a balanced diet and daily consumption of calories among the working-class population in India. The data was collected in Pune, India. It also studies the use of health apps in India and their impact on following a balanced diet and daily calorie intake. The data were analyzed using MS Excel and JASP software. The demographic profile of respondents is as follows: the number of males and females in the survey were 264 and 136, respectively. All respondents were graduates and above. The income level of respondents was as follows: 130 respondents were recorded in Indian Rupees (1 Lakh = 100,000), Rs. 1–5 Lakhs, 161 respondents in the income group of Rs. 5–10 Lakhs and the remaining 109 were in the income group above Rs. 10 Lakhs per annum. The age group of respondents was as follows: 266 respondents were in the age group of 25–40 years, 79 respondents in the age group of 41–55 years, and the remaining 55 were above 55 years of age.

### 4.2 Declarative knowledge

To check declarative knowledge, we used a trichotomous scale [28]. 239 respondents do not know their daily calorie consumption, 88 are not sure and only 73 are aware of their calorie intake. 285 are not aware of their recommended daily calorie intake. This shows that the majority working population lacks declarative knowledge about the calorie intake of food consumed daily. Regarding a balanced diet, 221 are aware of a balanced diet, 103 know something about a balanced diet, and 76 are not aware of a balanced diet. This shows that people have a declarative knowledge about a balanced diet but, when digging deeper into a balanced diet’s five food groups, the results were different. 195 do not know five food groups.74 know something and only 131 were aware of 5 food groups in a balanced diet. Again, in the case of nutrients in a balanced diet, 128 know the nutrients in a balanced diet, 94 know something and 182 do not know anything about nutrients. 157 were able to able to write the name of nutrients. 243 did not know anything about nutrients in a balanced diet. This shows people in India lack declarative knowledge about a balanced diet and daily calorie intake.

### 4.3 Exploratory factor analysis (EFA)

An exploratory factor analysis was executed to verify the factors underlying the consumer’s attitude. The adequacy of the sample was tested and also tested was whether the data permitted factor analysis. A Kaiser-Meyer-Olkin (KMO) Sample Adequacy Measurement was used to test the adequacy of the sample. The figure for the KMO was 0.73. This shows that the sample was appropriate [58]. The Bartlett-Sphericity test was used to determine whether the data should be used for factor analysis or not. The data (Chi-square = 886.77; df = 33; significant at p ޒ 0.001) enabled the study of exploratory factor analysis, as recommended by [58]. EFA results are presented in Table 2.

**Table 2:** EFA results.

Results of Exploratory Factor Analysis					
Latent Variables	Factors	Items Mean	Standard Deviation	Factor loadings	Reliability analysis (Cronbach’s alpha)
**Beliefs**	F1	3.415	0.863	0.984	0.85
	F2	3.257	1.157	0.983	
**Emotions**	F3	1.603	0.729	0.865	0.78
	F4	1.605	0.732	0.855	
	F5	1.972	0.908	0.572	
	F6	1.61	0.738	0.535	
	F7	1.762	0.792	0.523	
	F8	1.69	0.721	0.572	
**Intentions**	F9	1.758	0.013	0.892	0.82
	F10	1.758	0.752	0.892	
	F11	2.575	0.843	0.574	
	F12	1.667	0.709	0.549	

(Source: own calculations).

Three factors extracted from the results are shown in Table 2. Items F1-F2 are associated with customer beliefs for a balanced diet and daily consumption of calories. This element was then referred to as ’beliefs.’ Items F3-F8 clarified the feelings that customers maintained balanced nutrition and an intake of calories every day; thus the element was known as ‘emotions’. Except for F9, the points F10-F12 demonstrated the consumer’s intentions about the balanced diet, hence the factors were referred to as ‘intentions’. Item F9 was removed since only one of the three variables was able to load it. Instead of a belief, items F3 and F4 were translated as emotions. Cronbach’s alpha was 0.857 as indicated in Table 2 [54, 55].

### 4.4 Confirmatory factor analysis (CFA)

Validity of the dimensions of customer attitude was assessed after extraction of three variables by CFA; namely beliefs, intentions, and feelings. JASP software conducted confirmatory factor analysis. The results are shown in Table 3.

**Table 3:** CFA results.

Model Fit Indices	
Measurement	Index
Sample size	400
Chi-Square (CMIN)	45.942
Degrees of freedom(df)	32
CMIN/df	1.435
Comparative fit index	0.978
Root mean square error of approximation (RMSEA)	0.995

(Source: own calculations).

The model fit indices were acceptable as per Table 3 (Tukey-Lewis index (TLI)) = 0.992). F3 and F9 were perfectly correlated, F4 and F10 respectively. So were deleted by confirmatory factor analysis (CFA). The choice to agree on these indices is grounded on the references of [56, 57] that GFI and TLI should be close to 1 and CMIN/df should be less than 3.

**Fig 4:**
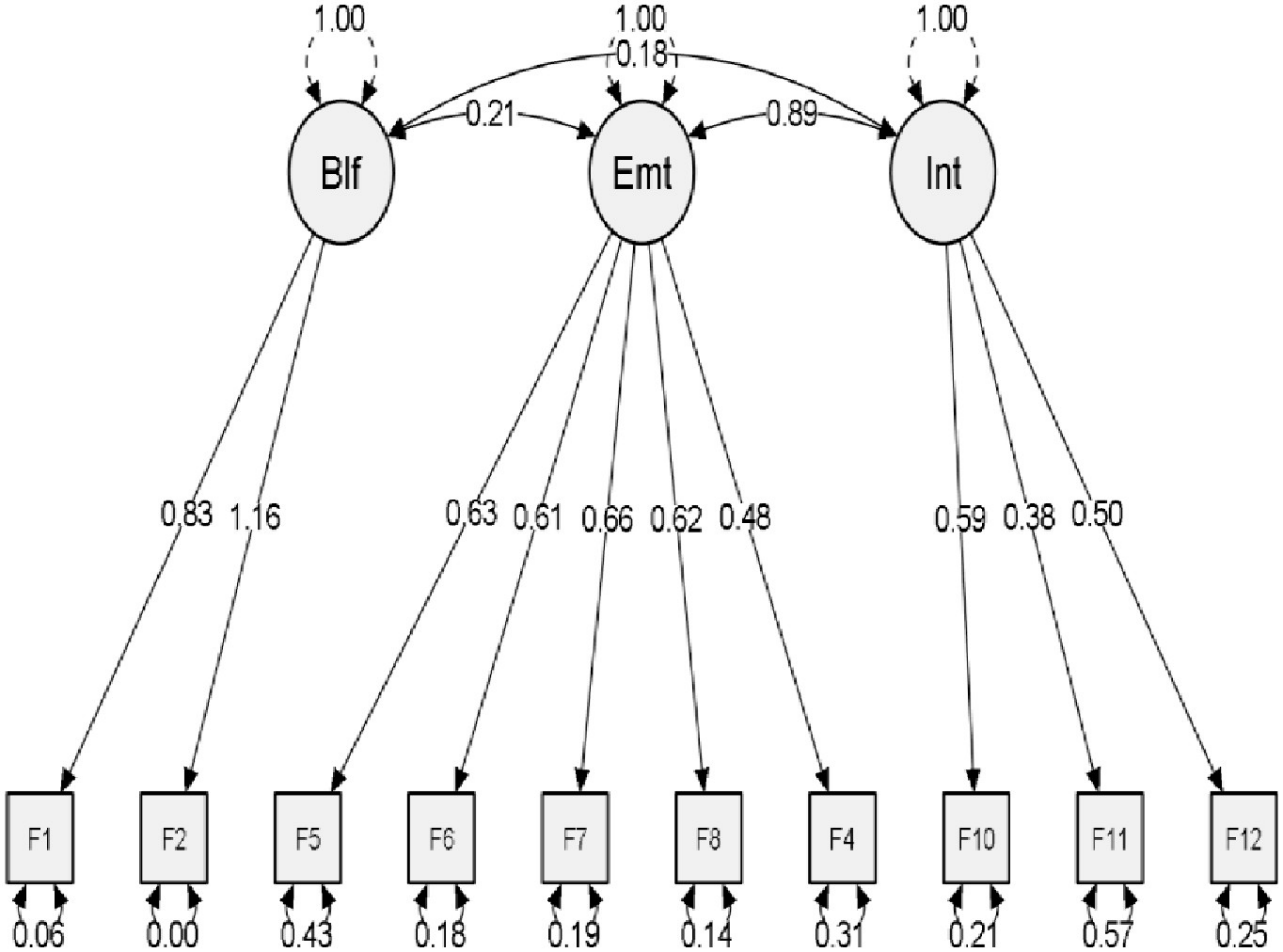
SEM model. (Source: own calculation).

A structural model was developed, and the estimates of the model were measured to calculate their significance. The items loaded (p<0.001) with latent variables were sufficient to be greater than 0.4, as shown in Fig 4 [58]. There were positive correlations between latent variables. It was determined that the association between beliefs and emotions is 0.18; p is significant (p < 0.001). The association between feelings and intentions is found at 0.89. It was determined that 0.20 is the association between beliefs and intentions (p < 0.001). These results show that latent variables are convergent. From Table 4, all hypotheses are accepted. Hypothesis H1 and H2 show that a balanced diet and monitoring daily calorie consumption are positively associated with the benefit of good health. Hypothesis H3 shows that monitoring daily calorie intake is positively associated with reducing the threat of infection of COVID-19. Hypothesis H4 and H5 show that consumption of a balanced diet and monitoring calorie consumption is related to happiness. Hypothesis H6 and H7 show that consuming a balanced diet and monitoring calorie consumption is related to a stronger self in the COVID-19 pandemic. Hypothesis H8 shows that following a balanced diet, as a necessity, is positively associated with intentions due to the threat of COVID-19. Hypothesis H9 and H10 show that recommendation to others to follow a balanced diet and monitor daily calorie intake is positively associated with intentions due to the threat of the COVID-19 pandemic.

**Table 4:** Factor loadings.

Factor loadings						
Factor	Indicator	Estimate	Std. Error	z-value	p	Hypothesis
**Beliefs**	F1	0.83	0.044	18.884	< .001	Accept H1
	F2	1.16	0.059	19.641	< .001	Accept H2
**Emotions**	F5	0.63	0.041	15.374	< .001	Accept H5
	F6	0.61	0.031	19.534	< .001	Accept H6
	F7	0.66	0.033	20.165	< .001	Accept H7
	F8	0.62	0.03	20.87	< .001	Accept H8
	F4	0.48	0.034	14.151	< .001	Accept H4
**Intentions**	F10	0.59	0.035	17.009	< .001	Accept H10
	F11	0.38	0.043	8.693	< .001	Accept H11
	F12	0.5	0.034	14.907	< .001	Accept H12

(Source: own calculations).

## 5. Discussion of results

The consumer attitude of people during the COVID-19 pandemic was studied using the tri-component attitude model. The purpose of the research was to evaluate the validity, reliability, and dimensionality of consumers’ attitudes to a balanced diet and daily consumption of calories during the COVID-19 pandemic in a developing country. As several authors have indicated (28,30), the tri-component attitude model of behavior; which studies belief (cognition), emotions (affect), and intention to follow (conation), have been proven as true and reliable customer attitude measures in an emerging economy. This means that consumer emotions, beliefs, and intent are key considerations in the COVID-19 pandemic that affects consumer decisions.

The vital association of consumer feelings and buying intentions reveal that consumers are more prepared to adopt a balanced diet when their emotions are favored. Consumer belief is also positively linked to follow-up intentions. This means that customer decisions to maintain a balanced diet and control the daily intake of calories will also be affected by consumer beliefs of a balanced diet. The connections between customer beliefs, emotions, and purchasing intentions show convergence; i.e., these components converge on the mindset of the consumer but, since the correlations are not high, Indian consumers still see these components as distinctive.

The cognitive component studied the declarative knowledge of a balanced diet and daily calorie consumption among people. While most people were aware of a balanced diet, the majority were not aware of their daily calorie consumption. The knowledge of a balanced diet is limited to name only as a majority were not able to name the five foods in a balanced diet. In addition, the majority were unaware of the nutrients in a balanced diet. As people are not aware of their daily calorie consumption, it may result in an intake of more calories, which may be harmful to their health. It may increase chronic and non-chronic diseases. From the data collected, people in the age group of 25–40 hardly have any health issues but people above age the age of 40 suffer from various health issues like diabetes, blood pressure, thyroid problems, arthritis, etc. Therefore, it is the responsibility of the government and corporate world to create awareness about a balanced diet and daily calorie intake through social awareness campaigns. Food companies can develop innovative campaigns [59, 60] and products through corporate social responsibility that help change people’s attitudes towards a balanced diet and daily calorie intake.

### 5.1 Implications for industry and government

Companies in food production need to be more research-oriented for products with components that are not suitable from a balanced diet point-of-view. For example, wheat flour-based items can be substituted with whole wheat-based items, and wheat can also be complemented or substituted by other grains like millets in packaged food products. Companies can have their products listed in diet apps for consumer awareness about the composition of food items. Consumers have an average awareness about a balanced diet but are not exposed to any appropriate measuring method. They need to be made aware of the measurements. A mobile app can play a significant role here. The Indian consumer is a food-loving consumer. Traditional or modern foods that are calorie-rich can be substituted without changing the palate significantly. For example, the introduction by food brand Maggi of multi-grain products or oats has been very well received by Indian consumers. Packaged food products have a high amount of preservatives that can be substituted with natural preservatives. Consumers can demand these if they are aware of such substitutes. The change can be introduced from a production perspective if the change is demanded from the consumption side. Therefore, consumers have to demand healthy changes in their foods; especially packaged food, and that requires knowledge about a balanced diet and monitoring daily calorie intake.

Results from the experiment to increase awareness of a balanced diet have been fruitful. A. Alam found that the balanced plate intervention helped women through practical demonstration to learn about a balanced meal by highlighting appropriate portion sizes and food diversity [61]. Innovative solutions [62, 63] can be developed to create awareness about a balanced diet and daily calorie intake. Social media can be used by corporate organizations and local governments to promote a balanced diet and to monitor daily calorie consumption [64]. Governments should undertake social marketing to promote the importance of consuming a balanced diet and monitoring daily calorie intake, which is rarely seen in a developing country like India. Governments should take the initiative for a public-private partnership to promote healthy eating behaviors through social advertising.

## 6. Conclusion

The conative, cognitive, and affective components explain the respondents’ attitudes towards a balanced diet and monitoring daily calorie consumption. The model helps to understand declarative knowledge about a balanced diet and daily calorie consumption. People are found lacking declarative knowledge about a balanced diet and daily calorie consumption. Relatively, females are much more aware and have the intention to follow balanced diet. Nutrition professionals and government agencies should work with local leaders and retired people to provide complete dietary knowledge to the working population. Encouraging parents and caretakers to purchase and make healthy foods regularly will go a long way to imbibe the culture of a balanced diet and daily calorie consumption among the working population. The working population and community at large are a valuable component of any economy and children seem keen to receive and follow nutritional recommendation from their parents. Males who are less aware but seem receptive to subjective norms would benefit more from family and peers’ activities to increase healthy eating behaviors. Because of the alarming increase in recurring diseases like COVID-19 in India and abroad, it is prudent to promote healthy dietary behavior through knowledge dissemination about the importance of a balanced diet and daily calorie consumption. This will help to play down the fears about recurring diseases and push people’s intention to follow a balanced diet and monitor daily calorie consumption. This will help reduce the prevalence of obesity and other chronic diseases among the working population in India and further help to reduce the threat of COVID-19-like pandemics.

## 7. Limitations and future scope for research

Data was collected through an online questionnaire using Google Forms. Other options normally available for data collection (such as interviews) were not available due to the COVID-19 pandemic and subsequent lockdown in India. The convenience sampling method used may not be entirely representative of the population. The authors have used the tri-component attitude model, utilized to study dietary behaviour. The paper only studies the consumer attitude of the working population towards a balanced diet and daily calorie consumption. The research cannot be generalized to a larger population. Further studies can be carried out to study consumer attitudes of children, females, senior citizens towards a balanced diet and daily calorie consumption, separately. The efficacy of the tri-component attitude model can be tested on different age groups in India and other countries in this sector. Future researchers can study procedural knowledge about a balanced diet and daily calorie consumption. Longitudinal studies and experimental interventions can be undertaken to study such phenomena. To provide a robust understanding of estimation and dimensionality, future investigations can also be undertaken in other emerging economies.

## Supporting information

S1 Data (XLSX)
